# Single-Step Hydrothermal Synthesis of Biochar from H_3_PO_4_-Activated Lettuce Waste for Efficient Adsorption of Cd(II) in Aqueous Solution

**DOI:** 10.3390/molecules27010269

**Published:** 2022-01-02

**Authors:** Quyun Chen, Tian C. Zhang, Like Ouyang, Shaojun Yuan

**Affiliations:** 1 Low-Carbon Technology & Chemical Reaction Engineering Lab, College of Chemical Engineering, Sichuan University, Chengdu 610065, China; cquyun@163.com (Q.C.); like.ouyang@scu.edu.cn (L.O.); 2 Civil & Environmental Engineering Department, University of Nebraska-Lincoln, Omaha, NE 68182-0178, USA; tzhang1@unl.edu

**Keywords:** biochar, hydrothermal carbonization, H_3_PO_4_ activator, adsorption mechanisms, lettuce waste

## Abstract

Developing an ideal and cheap adsorbent for adsorbing heavy metals from aqueous solution has been urgently need. In this study, a novel, effective and low-cost method was developed to prepare the biochar from lettuce waste with H_3_PO_4_ as an acidic activation agent at a low-temperature (circa 200 °C) hydrothermal carbonization process. A batch adsorption experiment demonstrated that the biochar reaches the adsorption equilibrium within 30 min, and the optimal adsorption capacity of Cd(II) is 195.8 mg∙g^−1^ at solution pH 6.0, which is significantly improved from circa 20.5 mg∙g^−1^ of the original biochar without activator. The fitting results of the prepared biochar adsorption data conform to the pseudo-second-order kinetic model (PSO) and the Sips isotherm model, and the Cd(II) adsorption is a spontaneous and exothermic process. The hypothetical adsorption mechanism is mainly composed of ion exchange, electrostatic attraction, and surface complexation. This work offers a novel and low-temperature strategy to produce cheap and promising carbon-based adsorbents from organic vegetation wastes for removing heavy metals in aquatic environment efficiently.

## 1. Introduction

Heavy metals (with their density exceeding 5 g∙cm^−3^) are produced from different industries, such as industrial production, wastewater irrigation, and agriculture activities. Heavy metals in the environment present a potential hazard to the ecosystem, public health, and safety due to their toxicity and carcinogenicity [1,2,3,4]. Recently, many technologies for heavy metal purification—including precipitation, coagulation-flocculation, ion exchange, membrane filtration, electroplating, and adsorption—have been developed [1,5,6]. Among them, the adsorption has the features such as high removal efficiency, renewable adsorbent, and flexible design and operation, which is a promising technology for removing heavy metals [7,8]. Traditionally, carbon materials are preferred adsorbents for removing pollutants in wastewater [9], many strategies have been employed to prepare activated carbon by activating biomass waste at a high pyrolysis temperature.

Over the last few decades, biochars have been reported to possess a good adsorption capacity toward heavy metal ions, and the characteristics and structures of biochars play an important role in this process [10]. To obtain effective biochar adsorbents, various agricultural wastes and by-products have been explored, such as palmae shell [11], hardwood [12], corn straw [12], phragmites biomass [13], banana peels [14], mango peels [15], cauliflower leaves [16], etc. Over the past few decades, the pyrolysis method has usually been used to prepare biochars [17], which has a low product yield but needs high calcination temperature, high energy consumption, fast cracking reaction speed, and a large amount of coke. Awfully, a large amount of harmful gas would be produced in the process of calcination, causing serious air pollution and greenhouse gas (GHG) emissions [18]. Generally speaking, pyrolysis efficiency, product quality, and adsorption performance are related to the inherent characteristics of biomass raw materials with high moisture, oxygen and alkali metal content, and it requires a higher energy input and a pyrolysis reactor with higher corrosion resistance in this process. Thus, the results often fall short of expectations [19,20]. During the pyrolysis of biomass, a cross-linking reaction occurs, resulting in a lot of tar attached to the surface of absorbents, which is very likely to hinder the contact between the biochar and heavy metals.

Alternatively, it has been reported previously that the carbon obtained from hydrothermal carbonization is expected to be a better adsorbent [21,22]. For example, Zhou et al. [14] have proven that, through the hydrothermal carbonization (HTC) process, fresh banana peels could be activated with situ into effective sorbents by using acidic activation agent. Fernandez et al. [23] used orange peel to prepare biochar by hydrothermal carbonization. Compared with the traditional pyrolysis, the hydrothermal method has the advantages of low energy consumption, low reaction temperature, low CO_2_ emission, and no limitation to the moisture content of the raw material. However, sufficient information is not available on how to use the hydrothermal method and desired biomass with high moisture content as a raw material to make biochar, let alone the effects of acidic activating agents (such as H_3_PO_4_, HNO_3_, etc.) on physicochemical characteristics of the biochar. Vegetation waste is an ever-growing global problem [24]. Most of the methods used to deal with vegetation waste in the world still are very unscientific—such as random dumping, landfilling, stockpiling for accumulation of vegetation waste, etc.—which has caused a series of environmental, economic, and social problems, and calls for sustainable, cost-effective, and efficient methods to deal with these wastes. To our knowledge, there is a lack of studies which have been reported in the literature to develop biochar adsorbents with vegetation wastes for heavy metal removal.

**Scheme 1:**
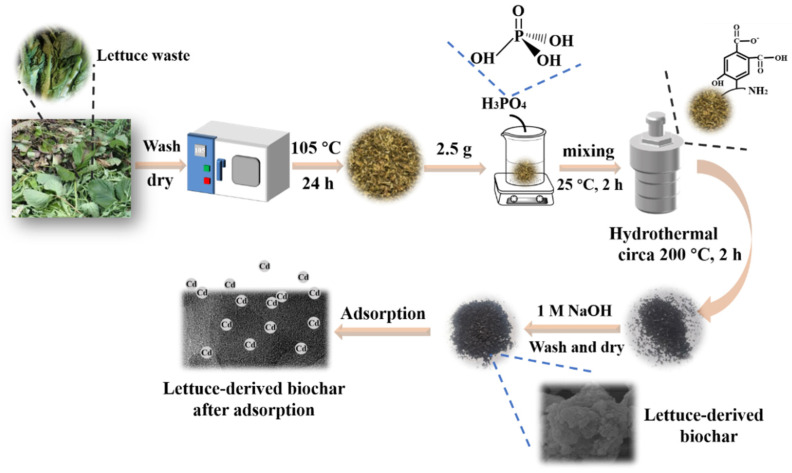
Schematic diagram illustrating a single-step hydrothermal synthesis of biochar from lettuce wastes and used as biochar adsorbent for adsorption of Cd(II).

Herein, this study aims at preparing biochar adsorbents, with lettuce waste as raw material, via a single-step hydrothermal carbonization for removing Cd(II) ions from aqueous solutions effectively. As schematic illustration in Scheme 1, hydrothermal carbonization was performed by using H_3_PO_4_ as an acidic activation agent. The optimal hydrothermal synthesis conditions—such as hydrothermal reaction time, hydrothermal reaction temperature, and the concentrations of H_3_PO_4_—were systematically explored to produce biochar from lettuce wastes. Furthermore, the impacts of different hydrothermal synthesis conditions on the physicochemical characteristics of biochar were also investigated by the measurements of the Fourier transform infrared spectroscopy (FTIR) spectra, Raman spectra, X-ray diffraction (XRD) patterns, and thermogravimetric (TG) curves. Batch adsorption experiments were carried out to determine the influence of environmental factors including solution pH, initial concentration of Cd(II), and adsorbent dosage on sorption performance, sorption kinetics, sorption isotherms, and thermodynamic parameters of the biochar towards Cd(II). The competition adsorption was conducted on adsorption properties of H_3_PO_4_-activated biochar from lettuce waste for typical heavy metal ions of Pb(II), Cu(II), Zn(II), and Cd(II). The adsorptive mechanisms toward Cd(II) on the biochar was proposed and verified by XPS characterization and zeta potentials testing.

## 2. Results and Discussion

### 2.1. Determination of Optimal Hydrothermal Conditions of H_3_PO_4_-Activated Biochar

**Figure 1:**
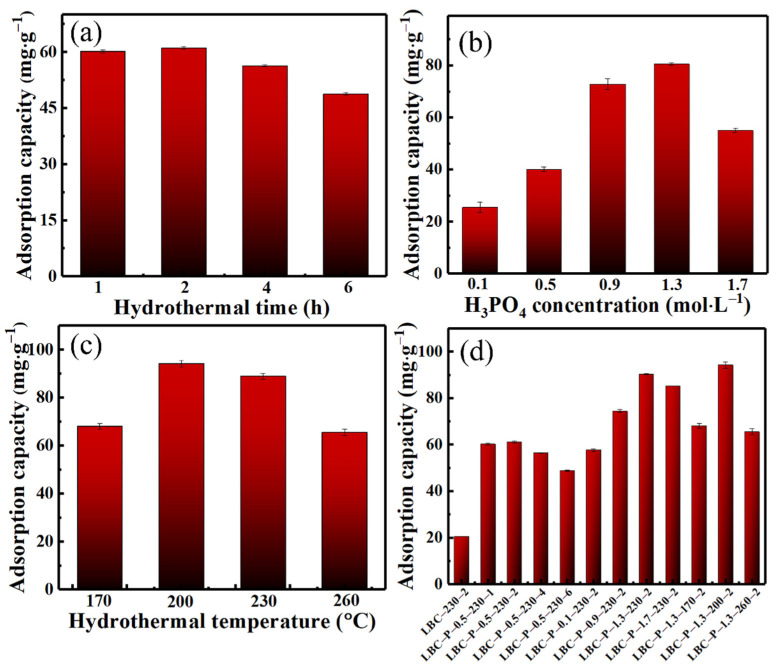
Effect of different hydrothermal synthesis conditions on the H_3_PO_4_-activated biochar towards the adsorption capacity of Cd(II) ions: (**a**) hydrothermal reaction time (Table S1, hydrothermal reaction temperature at 230 °C, and H_3_PO_4_ concentration of 0.5 mol∙L^−1^), (**b**) the H_3_PO_4_ concentration of (Table S2, hydrothermal reaction temperature at 230 °C, and hydrothermal reaction time at 2 h), and (**c**) hydrothermal reaction temperatures (Table S3, H_3_PO_4_ concentration of 1.3 mol∙L^−1^, and hydrothermal reaction time at 2 h), (**d**) the comparison of Cd (II) adsorption capacity by different biochar samples. Batch adsorption experimental conditions: pH = 6.0, C_0_ = 100 mg∙L^−1^, T = 25 ± 1 °C, m = 1.0 g∙L^−1^, v = 50 mL, t = 100 min.

Different conditions—including hydrothermal reaction time, temperatures, and the addition concentrations of H_3_PO_4_ activation agent—were chosen to determine the optimal hydrothermal synthesis conditions of biochar with the addition of H_3_PO_4_ activation agent, and detailed information of hydrothermal synthesis were shown in Tables S1–S3. Figure 1 displayed the adsorption capacities of Cd(II) on the H_3_PO_4_-activated biochar obtained with different synthesis conditions. For the biochar derived from hydrothermal reaction time, the sample obtained by hydrothermal reaction of 2 h exhibited the highest adsorption capacity at approximately 61.4 mg∙g^−1^, while further increasing hydrothermal reaction time, the adsorption capacity underwent a markable drop to approximately 12.3 mg∙g^−1^ (Figure 1a). It could be seen from Figure 1b that the adsorption capacities of biochars obtained at different activator concentrations increased with an increase in the concentration of H_3_PO_4_ activating agent. The highest adsorption capacity of such biochar increased to circa 90.2 mg∙g^−1^ with an activator concentration at 1.3 mol∙L^−1^. Further increasing the H_3_PO_4_ activator concentration to 1.7 mol∙L^−1^ results in the adsorption capacity of the obtained biochar decreasing slightly (Figure 1b). For the effect of hydrothermal reaction temperature on the adsorption capacities of as-prepared biochar, the hydrothermal temperature at lower than 200 °C was not conducive to the formation of good lettuce waste-based biochar (Figure 1c). The optimal hydrothermal temperature to synthesize biochar was determined at 200 °C. Taken together, the optimal hydrothermal synthesis conditions of biochar from lettuce wastes were determined as the follows: hydrothermal reaction time at 2 h, the H_3_PO_4_ activator concentration of 1.3 mol∙L^−1^, and hydrothermal temperature at 200 °C. As shown in Figure 1d, under optimal hydrothermal synthesis conditions, the as-fabricated LBC-P-1.3-200-2 biochar sample exhibited a equilibrium adsorption capacity of Cd(II) at circa 94.2 mg∙g^−1^. Therefore, the as-fabricated LBC-P-1.3-200-2 biochar was chosen for subsequent characterization and adsorption testing.

### 2.2. Characterization of H_3_PO_4_-Activated Biochar

**Figure 2:**
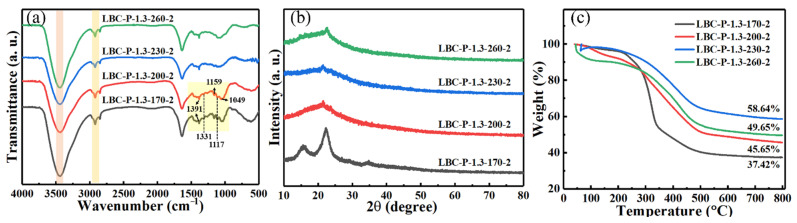
(**a**) FT-IR spectra, (**b**) XRD patterns, and (**c**) TG curves of LBC-P-1.3-X-2 (X = 170, 200, 230, and 260 °C) samples.

Figure 2a showed the FTIR spectra of different H_3_PO_4_-activated biochars at various hydrothermal reaction temperatures, respectively. The corresponding FTIR spectra of the H_3_PO_4_-activated biochar obtained at different hydrothermal reaction time and activator concentrations were displayed in Figures S1a and S2a, respectively. Similar characteristic peaks were observed in the FTIR spectra of these biochar simples as shown in Figure 2a, Figures S1a and S2a. With an increase in hydrothermal reaction temperature (Figure 2a) and time (Figure S1a), some peaks within 1000–1500 cm^−1^ were more difficult to distinguish. The main characteristic FTIR bands were observed to include −OH, P=O, C−O, C−C, and C−N groups [9,25,26]. The stretching vibration of O−H in the hydroxyl groups and carboxyl groups leads to the appearance of peaks at 3454 cm^−1^ and 1641 cm^−1^, respectively [27]. The peak at 1049 cm^−1^ was related to the bonds of P=O, while the band 1117 cm^−1^ was assigned to C−O, C−X, or C−C stretching vibration. The peak at 1159 cm^−1^ was attributed to C−O in phenol, whilst the peak at 1391 cm^−1^ could be ascribed to the stretching vibration of C=N or C−N. On the other hand, with increasing the H_3_PO_4_ activator concentration, the peaks at 500–1500 cm^−1^—such as P=O and C−N—stretching vibration increased (Figure S2a). The FTIR spectra suggested the existence of nitrogen and phosphorus elements on the biochar materials activated by H_3_PO_4_. The XRD patterns of different H_3_PO_4_-activated biochars at different hydrothermal reaction temperatures, reaction time, and activator concentrations were showed in Figure 2b, Figures S1b and S2b, respectively. All H_3_PO_4_-activated biochar samples revealed a weak diffraction peak at 22.5°, indicting the amorphous feature of these biochar samples [28].

In TG testing, an aliquot 0.01 g of sample was placed in an Al_2_O_3_ ceramic crucible in N_2_ atmosphere, and the sample was heated from 20 to 800 °C with a heating rate of 10 °C/min. The TG curves of different biochar samples were shown in Figure 2c, Figures S1c and S2c, respectively, to provide useful information for the thermal stability of all biochar samples. Obviously, all these biochar samples activated by H_3_PO_4_ had the same weight loss trend. These biochar simples exhibited three weightless stages (Figure 2c). The first stage of weight loss was ascribed to the evaporation of water adsorbed on the biochar surface, which resulted in approximately 5% of weight loss of at 140 °C except for the LBC-P-1.3-260-2 sample with a weight loss of circa 10%. The second stage was associated with the decomposition of some volatile organic compounds [29]. When the temperature reached to 400 °C, the decomposition efficiency of LBC-230-2 and LBC-P-1.3-200-2 were up to approximately 41.01% and 29.62%, respectively. The H_3_PO_4_ activating agent seemed to prevent the volatilization of organic matter in the raw materials to varying degrees. The weight loss of approximately 20% in the third stage within the 400–535 °C range. In this stage, the hydrothermally carbonized biomass and biomass components such as lignin would be decomposed, resulting in further weight loss. Therefore, it can be inferred that the hydrothermally-synthesized biochar retained a large amount of organic matter in the lettuce waste and the addition of phosphoric acid can improve the thermal stability of biochar. In addition, appropriately increasing the hydrothermal time and hydrothermal temperature can also improve the stability of the biochars.

**Figure 3:**
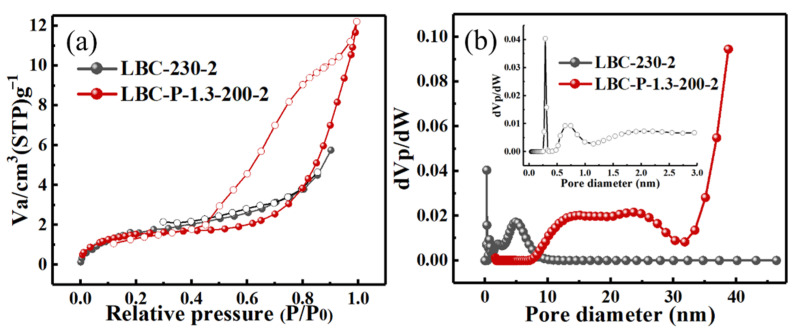
(**a**) N_2_ adsorption–desorption isotherms and (**b**) corresponding pore size distribution profiles of LBC-230-2 and LBC-P-1.3-200-2 samples.

To further determine the pore structure properties of the sample materials, N_2_ adsorption–desorption experiments were carried out on the biochar samples obtained by hydrothermal carbonization without and with H_3_PO_4_ activation. Figure 3a showed the N_2_ adsorption–desorption isotherms of tested samples at a temperature of 77 K, whilst Figure 3b represented the pore size distribution (PSD) curves for all the tested samples, which was determined by the BJH method. The adsorption–desorption isotherms of the biochars both belonged to type IV adsorption isotherms, of which, the hysteresis loop of LBC-230-2 was classified as H3 types, whilst that of LBC-P-1.3-200-2 returned H2 (b) (IUPAC classification) [30]. More comparison of the pore features of the biochar samples was performed as shown in Table S4. In comparison with the inactivated LBC-230-2 biochar, no microporous structure was observed in the H_3_PO_4_-activated LBC-P-1.3-200-2 biochar, which may be caused by the damage of pores with phosphoric acid activation. Furthermore, for LBC-P-1.3-200-2, a large number of mesopores and macropores was observed, which may be conducive to the adsorption of Cd(II). The surface areas of the hydrothermal biochar materials were not well-developed at low temperature (Table S4), which was a drawback of almost all the carbon materials synthesized by hydrothermal carbonization method.

**Figure 4:**
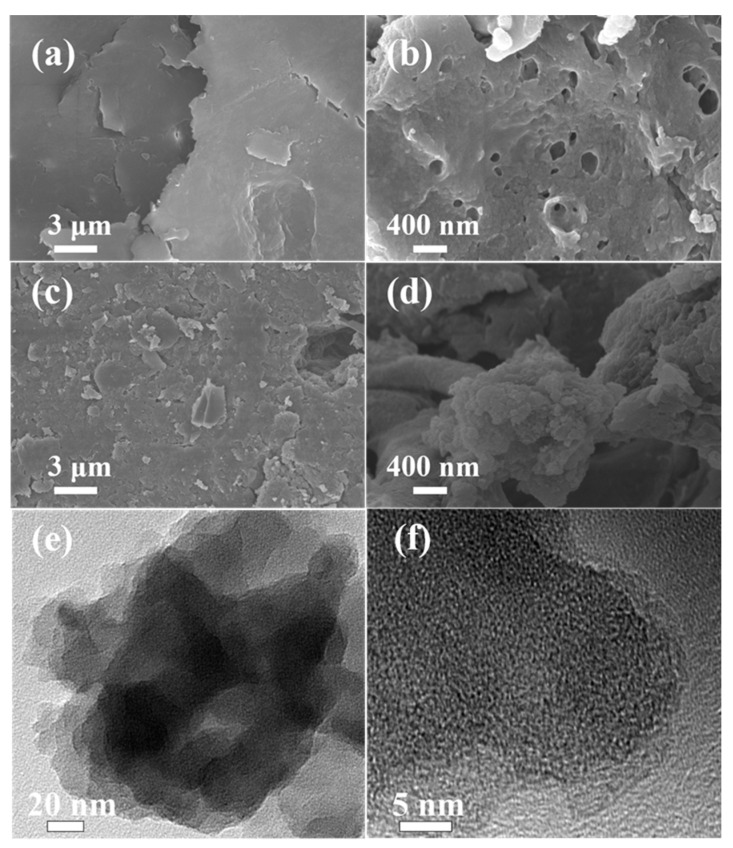
SEM pictures of: (**a**,**b**) LBC-230-2 and (**c**,**d**) LBC-P-1.3-200-2. (**e**,**f**) TEM images of LBC-P-1.3-200-2.

Figure 4 showed the representative SEM and TEM images of the LBC-230-2 (without activator addition) and LBC-P-1.3-200-2 samples. The LBC-230-2 surface showed a relatively smooth surface, and only sparse pores were spotted over the biochar materials (Figure 4a,b). After being activated by H_3_PO_4_ activator, the derived LBC-P-1.3-200-2 biochar samples exhibited a rougher surface and porous structure of amorphous carbon materials [31]. TEM imaging further confirmed the amorphous structure of the LBC-P-1.3-200-2 biochar sample (Figure 4e), and the highly disordered and low crystallinity carbon was also revealed from the high-resolution TEM images (Figure 4f).

**Figure 5:**
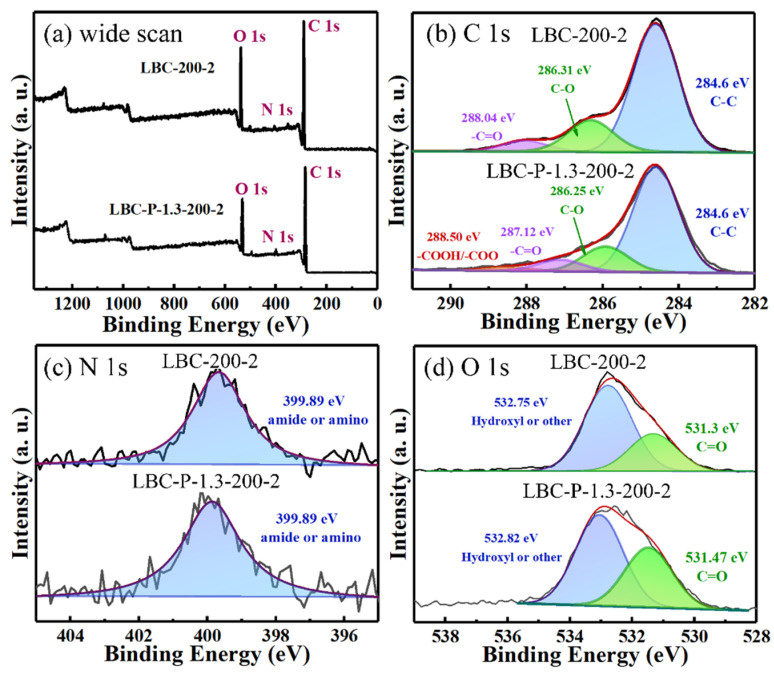
The XPS spectra of (**a**) wide scan, (**b**) C 1s, (**c**) N 1s, and (**d**) O 1s for the LBC-200-2 and LBC-P-1.3-200-2.

As shown in Figure 5, XPS spectra of inactivated biochar and H_3_PO_4_-activated biochar were obtained by XPS measurement, and the effect of H_3_PO_4_ activator on the surface chemistry of biochar samples was further studied. The wide scan spectrum of LBC-P-1.3-200-2 sample showed four peak signals with binding energies (BEs) at 298.3, 410.3, and 545.3 eV, ascribed to C 1s, N 1s, and O 1s signals, respectively. The corresponding elemental contents were shown in Table S5. The curve-fitted C 1s core-level spectra of the two biochar samples displayed similar peak components. The BEs at 284.6, 286.31 (286.25), and 287.12 (288.04) eV, attributed to the C−C, C−O, and −C=O, respectively [32], except that an additional minor peak at 288.5 eV appeared, attributable to the carboxyl groups (O=C−O^−^) of the LBC-P-1.3-200-2 sample (Figure 5a). This result suggested the acidification of biochar in a H_3_PO_4_-activation hydrothermal synthesis process. No difference could be distinguished from the deconvoluted N 1s core-level spectra of the two biochar samples, which displayed the amide or amino groups (399.89 eV) (Figure 5c). The deconvoluted O 1s core-level spectrum of LBC-200-2 and LBC-P-1.3-200-2 samples both showed two peak components with BEs at circa 532.8 and 531.5 eV, corresponding to the hydroxyl and C=O (Figure 5d), respectively [33]. The relative abundance of C=O in the LBC-P-1.3-200-2 sample increased significantly, indicating that the activation of lettuce waste by H_3_PO_4_ influences the local chemical states of O in hydrothermal synthesis.

### 2.3. Effect of Environmental Factors on Adsorption Performance

**Figure 6:**
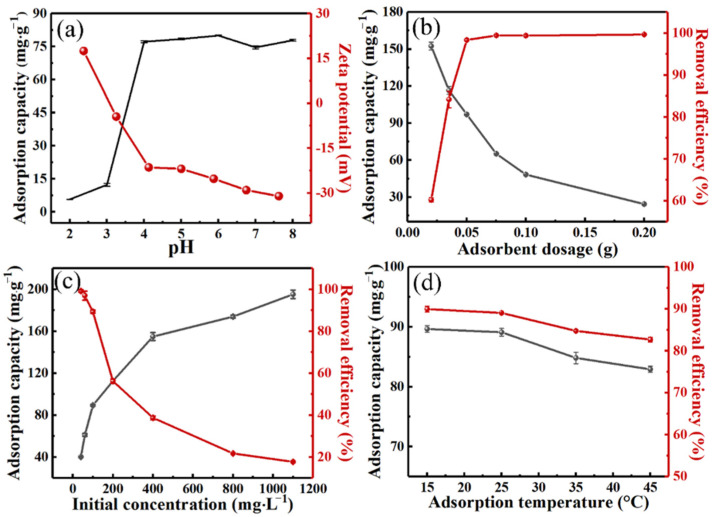
Effect of different environmental factors on the Cd(II) adsorption capacity on the LBC-P-1.3-200-2 biochar sample: (**a**) initial solution pH and zeta potentials, (**b**) adsorption dosages, (**c**) initial concentrations of Cd(II), and (**d**) adsorption temperature. Batch experimental conditions: T = 25 ± 1 °C, C_0_ = 100 mg∙L^−1^, m = 1.0 g∙L^−1^, v = 50 mL, t = 100 min, pH = 6.0.

The initial pH is an important control parameter. The adsorption capacity of Cd(II) under different pH (2–8) and the result of the zeta potentials test were displayed in Figure 6a. In general, the adsorption capacity of LBC-P-1.3-200-2 for Cd(II) increased as the pH increased, but was very low at pH 2. The cationic molecules would be competed for adsorption sites with the protonation of Cd(II) in acidic media and the excess H ion. At a very low pH, the surface ligand bound closely to the hydronium ion (H_3_O^+^), and the repulsive force restricted the approach of metal ions. Importantly, although most of the carboxyl groups participated in the complexation reaction, they would not be free at low pH and could not combine with the metal ions in the solution. Combined with the zeta potential results, the point of zero charge was approximately 2.8, which explained why at pH 3, the adsorption capacity increased sharply to approximately 78.76 mg∙g^−1^. Within a lower pH region, the LBC-P-1.3-200-2 had a positively-charged surface, and the electrostatic repulsion between biochar and Cd(II) ions was strengthened, resulting in a decrease in removal efficiency [34]. Therefore, the electrostatic interaction between LBC-P-1.3-200-2 and Cd(II) cation was the reason for its stable adsorption of Cd(II) and high capacity at the pH of 3–8. After the pH was higher than 7, there were a lot of OH^−^ ions in the solution, and the main adsorption mechanism was that OH^−^ and Cd(II) ions form Cd(OH)_2_ precipitation. Thus, the optimal solution pH was chosen as 6.0.

The effects of other factors, such as biochar dosage on the adsorption performance of the as-fabricated biochar sample, were further determined in batch adsorption experiment. The Cd(II) adsorption capacity was 97.32 mg∙g^−1^ and the removal efficiency of Cd(II) ions was close to 98.69% when LBC-P-1.3-200-2 of 0.05 g was used as the adsorbent. Continuing to increase the amount of LBC-P-1.3-200-2 adsorbent, the adsorption capacity was decreased dramatically, while the removal efficiency of Cd(II) did not increase significantly (Figure 6b). Therefore, an aliquot of 0.05 g biochar adsorbent dosage was used to ensure full utilization rate of the adsorbent in subsequent adsorption experiments. Moreover, the increase in Cd(II) initial concentration has a significant impact on the adsorption capacity and removal efficiency of the LBC-P-1.3-200-2 sample (Figure 6c). When the Cd(II) solution of 100 mg∙L^−1^ was used, the adsorption capacity of Cd(II) is approximately 88.47 mg∙g^−1^, and the removal efficiency is approximately 89.01%. Further increasing the initial concentration of Cd(II) resulted in sharp reduction in removal efficiency. In addition, the adsorption capacity of LBC-P-1.3-200-2 adsorbent seemed have a marginal effect on the adsorption process, albeit that the adsorption capacity of Cd(II) decreased slightly as the adsorption temperature increased from 15 °C to 45 °C (Figure 6d). Therefore, the optimal batch adsorption conditions for the LBC-P-1.3-200-2 biochar sample were determined as follows: solution pH at 6.0, adsorbent dosage of 0.05 g, initial concentration of Cd(II) at 100 mg∙L^−1^ and ambient adsorption temperature.

### 2.4. Adsorption Kinetics

**Figure 7:**
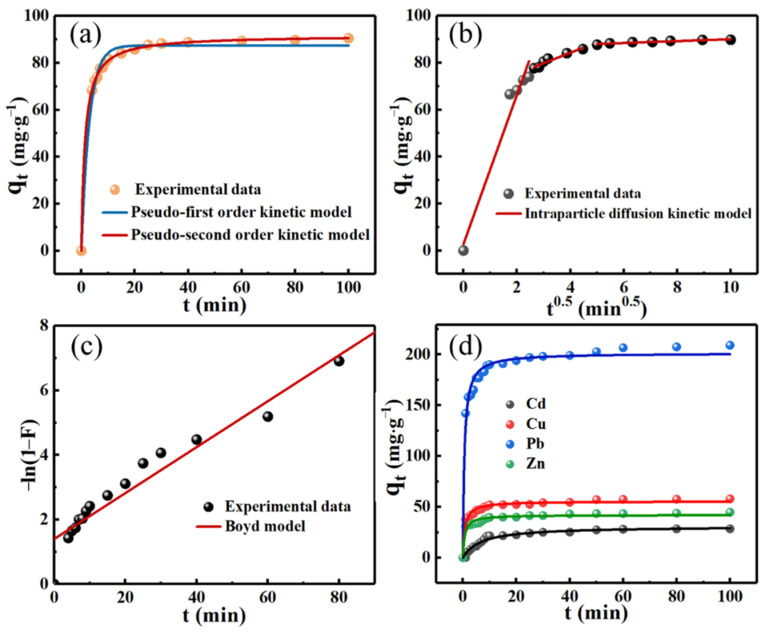
Adsorption kinetics of Cd(II) on the LBC-P-1.3-200-2 biochar and data fitting with (**a**) the pseudo-first order and pseudo-second order model, (**b**) Weber–Morris model, and (**c**) Boyd model. (**d**) Adsorption kinetics for Cd(II), Cu(II), Zn(II), and Pb(II) on the LBC-P-1.3-200-2 and data fitting with a pseudo-second order model. Batch experimental conditions: T = 25 ± 1 °C, C_0_ = 100 mg∙L^−1^, m = 1.0 g∙L^−1^, v = 50 mL, t = 100 min, pH = 6.0.

Adsorption kinetics was carried out to describe removal efficiency, which would be determined by the physical and chemical properties of the biochar and the mass transfer of heavy metals during the adsorption process [35]. Adsorption kinetic experiments were conducted under optimal adsorption conditions, and using the experimental kinetic data of Cd(II) to fit the adsorption kinetic models (S1.6). The information about the mechanisms and process of adsorption to the adsorbent was provided by adsorption kinetic models. Figure 7a showed the adsorption kinetics of Cd(II) on the LBC-P-1.3-200-2 sample fitted by the pseudo-first-order (PFO) and pseudo-second-order (PSO) kinetic models. The adsorption rate was quite fast in the initial 20 min. It is estimated that the adsorption amount in the first 20 min accounted for circa 94% of the total adsorption amounts. It was easy to conclude that the adsorption equilibrium of Cd(II) on the biochar samples was achieved within circa 30 min, and the equilibrium adsorption capacity was circa 90.48 mg∙g^−1^. This result was consistent with that the biochar had a fast adsorption rate for Cd(II) ions. The good fitting of the PSO model was observed, and the fitted kinetic model parameters of k_f_, k_s_, and q_e_ were displayed in Table 1. The equilibrium adsorption capacity of Cd(II) ions obtained by PSO model fitting was 91.75 mg∙g^−1^, did not demonstrate much difference from the actual adsorption capacity. In general, the adsorption process of Cd(II) ions by the LBC-P-1.3-200-2 was more consistent with the PSO model (R^2^ = 0.999). The results indicated that the adsorption kinetics was mainly controlled by chemical action. In other words, the Cd(II) ions reaching the surface of LBC-P-1.3-200-2 biochar from the solution was controlled by the chemical adsorption mechanism.

In order to further consider the whole adsorption process (mainly including several steps such as diffusion outside the membrane, diffusion inside the particle and chemical adsorption), the Weber–Morris and the Boyd models were used to fit the experimental adsorption kinetic (S1.6). For the adsorption kinetics of Cd(II) on the LBC-P-1.3-200-2, Figure 7b showed the fitted plots of the intra-particle diffusion model and Figure 7c showed the diffusion model with external liquid film. The fitted diffusion parameters were listed in Table 1. The Weber–Morris model presents a fitted-result with multi-linearity, indicating that the adsorption process of Cd(II) on the LBC-P-1.3-200-2 included more than one kinetic phase (Figure 7b). The values of the intercept C_id_ of these three linear parts were positive, indicating that the intra-particle diffusion might be a rate-controlling step for the diffusion rate. Besides, a certain degree of boundary layer diffusion control was also existed. The R-squared (R^2^) value of Boyd kinetic model (Figure 7c) was not satisfactory. It showed that boundary layer diffusion was only one of the rate-controlling steps. Therefore, these two diffusion models had a comprehensive influence on removing Cd(II) on LBC-P-1.3-200-2.

In a system with multi-component wastewater, the adsorption efficiency would be influenced by the coexisting metal ions. In this study, according to the result of actual investigation of the types of heavy metal ions in wastewater, four typical heavy metals (Pb(II), Cu(II), Zn(II), and Cd(II) ions) were selected as the co-existing metal ions to study the competitive adsorption effect. Figure S4a showed the adsorption performance of these four heavy metal ions on the LBC-P-1.3-200-2. During the initial adsorption process, the adsorption rate was fast, which was ascribed to the abundant active sites. As the adsorption reaction progressed, the adsorption tended to reach the plateau. Figure 7d showed the PSO-fitted adsorption kinetics of Cd(II), Cu(II), Zn(II), and Pb(II) on the LBC-P-1.3-200-2 sample, while the PFO-fitted counterpart was shown in Figure S4b. The PSO model fitted the kinetic data better with a larger R^2^ value, meaning that the adsorption process involved electron sharing or electron transfer between the LBC-P-1.3-200-2 and heavy metal ions and would be controlled by the chemical adsorption mechanism.

In a system with multiple heavy metal ions, the radius of hydrated ions and the ionic charge are the important factors affecting the results of competitive adsorption. Through analyzing the results of competitive adsorption experiment, the as-prepared biochar delivered outstanding adsorption capabilities to these four heavy metals with an order of Pb(II) > Cu(II) > Zn(II) > Cd(II), and the corresponding maximum adsorption capacities were between 28.5 and 209 mg∙g^−1^ (Figure S4a). Usually, both the natures of metal ions and the interactions between biochar and heavy metals in solutions might cause this result. According to the Pauling’s electronegativity order, the affinity order is at Pb (2.3), Cu (1.9), Cd (1.69), and Zn (1.65). However, the adsorption result is opposite to the order of cation hydrated radius is at Pb (4.01Å), Cu (4.19Å), Cd (4.26Å), and Zn (4.30Å). The hydration radius is a factor in the migration resistance of the adsorbate in the solution. When competing for adsorption sites, the smaller the hydration radius, the smaller the mass transfer resistance, hence it has the advantage of being preferentially adsorbed. Among them, the adsorption of Pb(II) ions is the most impressive and stable, the main reason of which may be related to the smaller hydration radius and valence state. As a result, the adsorptive sites were easily occupied by the cations with higher electronegativity and smaller hydrated radius via coordination or chelation reaction.

### 2.5. Adsorption Isotherms

**Figure 8:**
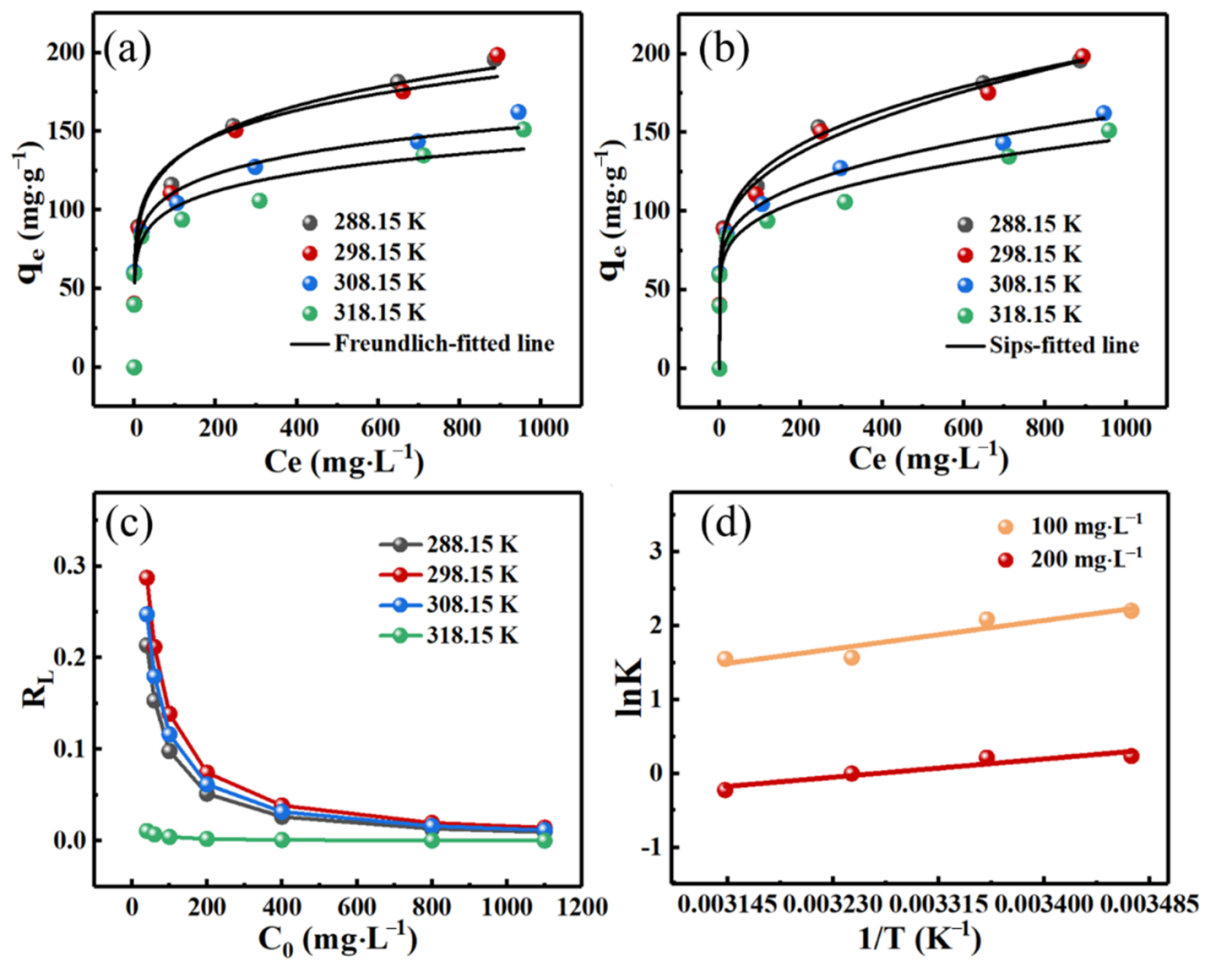
(**a**) Freundlich- and (**b**) Sips model-fitted adsorption isotherms of LBC-P-1.3-200-2 for Cd(II) at different adsorption temperatures of 288.15–318.15 K (**c**) The separation factor (*R_L_*) various with the initial concentration of Cd(II) ions on the LBC-P-1.3-200-2 at different temperatures. (**d**) Thermodynamic parameter analysis of Cd(II) removal on LBC-P-1.3-200-2. Batch adsorption experimental conditions: pH = 6.0, C_0_ = 40–1100 mg∙L^−1^, m = 1.0 g∙L^−1^, v = 50 mL, t = 100 min.

The adsorption capacity of the biochar can be evaluated by the adsorption isotherms, and the interaction between the Cd(II) ions and the biochar can be learned by the adsorption isotherms during the adsorption process [36]. Figure 8 showed the adsorption isotherms of Cd(II) ions on the LBC-P-1.3-200-2 at different adsorption temperatures from 288.15 to 318.15 K. The result showed that a higher removal efficiency was achieved at a lower concentration of Cd(II). When the Cd(II) concentration was increased to 1100 mg∙L^−1^, the equilibrium adsorption capacity of Cd(II) ions amounted to as high as circa 196.5 mg∙g^−1^. Moreover, the adsorption performance of Cd(II) decreased as the adsorption temperature increased, indicative of an exothermic adsorption process of Cd(II) ions on the as-prepared biochar sample.

Different adsorption isotherms—including Langmuir [37], Freundlich [38], Temkin [39], and Sips [40]—were used to fit the equilibrium experimental data. The corresponding formulas are presented in S1.7. The Langmuir model assumes the adsorption on a homogeneous surface through a single layer (Figure S4c) [41]. The assumption of the Freundlich model is to adsorb on a heterogeneous surface through multilayer adsorption (Figure 8a) [42]. The Sips model is a non-linear Freundlich–Langmuir isotherm (Figure 8b). If the value of n_s_ is equal to 1 or C_e_ or K_S_ approaches 0, then the Sips equation will become the Langmuir equation or the Freundlich isotherm, respectively. In addition, the obtained result of adsorption isotherms may be more realistic when the Sips model is used for fitting [43]. The Temkin model (Figure S4d) is a model for studying the linear relationship between the heat of adsorption and the temperature, and it is suitable for the chemical adsorption process in which the heat of adsorption changes linearly with the degree of surface coverage. The obtained adsorption constants and R^2^ were shown in Table 2, which, together with Figure 8, demonstrate that the Sips model was better-fitted than the other three adsorption isotherm models, suggesting the adsorption of Cd(II) on the heterogeneous surfaces of LBC-P-1.3-200-2. When the adsorption temperature was 298.15 K, q_m_ = 195.8 mg∙g^−1^, and R^2^ = 0.987 for the Sips model. In comparison with the previously-reported biochar samples from biomass, Table 3 summarized the q_m_ of biomass-based adsorbents obtained with different activating agents for Cd(II) removal. The q_m_ of Cd(II) on the functionalized biochar varied significantly from 1.5 to 472.1 mg∙g^−1^, such as H_2_SO_4_-activated bagasse pitch at 38.3 mg∙g^−1^ [44], H_3_PO_4_-activated chicken feather at 7.8 mg∙g^−1^ [45], Fe/Mn-modified biochar from *Pennisetum* sp. straw at 95.2 mg∙g^−1^ [46], succinic anhydride-modified biochar from microfibrillated cellulose at 236.1 mg∙g^−1^ [47], and so on [48,49,50,51,52]. The hydrothermally-synthesized biochar from H_3_PO_4_-activated lettuce waste in this study exhibited better adsorption performance of Cd(II). As a consequence, the biochar derived from vegetation waste via low-temperature hydrothermal synthesis may be feasible and cost-effective.

The values of separation factor constant (*R_L_*) can evaluate the degree of suitability of the LBC-P-1.3-200-2 sample towards Cd(II) ions, which shows the possibilities of the adsorption process to proceed: *R_L_* > 1.0 unsuitable, *R_L_* = 1.0 linear, 0 < *R_L_* < 1.0 suitable, and *R_L_* = 0 irreversible [53]

(1) $$R_{L}=\frac{1}{\left(1+K_{L}\times C_{0}\right)}$$

where *K_L_* is the Langmuir equilibrium constant (L∙mg^−1^), and *C*_0_ is the initial concentration of metal ions (mg∙L^−1^). Figure 8c presented the variation of *R_L_* as a function of the initial concentration of Cd(II) at different adsorption temperatures. The *R_L_* values lying between 0.01 and 0.29 indicated the suitability of the pristine adsorbents for Cd(II) removal from aqueous solution. Furthermore, with the increase of the initial concentration of Cd(II) and the adsorption temperature, the *R_L_* value gradually decreases to close to zero, indicative of unfavorable adsorption of Cd(II) ions on the LBC-P-1.3-200-2 sample surface at higher initial concentration of Cd(II) and higher adsorption temperatures.

### 2.6. Adsorption Thermodynamics

Adsorption thermodynamics were studied at adsorption temperatures of 288.15 to 318.15 K under optimal conditions. By calculating the enthalpy (ΔH°), entropy (ΔS°), and Gibbs free energy (ΔG°) from the Van’t Hoff diagram, the thermodynamic properties of the Cd(II) adsorption process on the LBC-P-1.3-200-2 biochar sample were obtained [54]. Detailed calculation procedures and equations were shown in S1.7. Figure 8d and Table 4 showed that ΔH° and ΔS° were both less than 0, together with ΔG°, meaning that the adsorption process of Cd(II) by the LBC-P-1.3-200-2 was a spontaneous and exothermic process. This result was basically the same with the aforementioned adsorption isotherms. It had been reported that the value of ΔG° was between −400 to −80 kJ∙mol^−1^ for chemical adsorption and −20 to 0 kJ∙mol^−1^ for physical adsorption [55]. The ΔG° values obtained in this study were within these two ranges, indicating that the adsorption process probably followed the ion exchange or surface complexation mechanism [54].

### 2.7. The Postulated Adsorption Mechanisms

**Figure 9:**
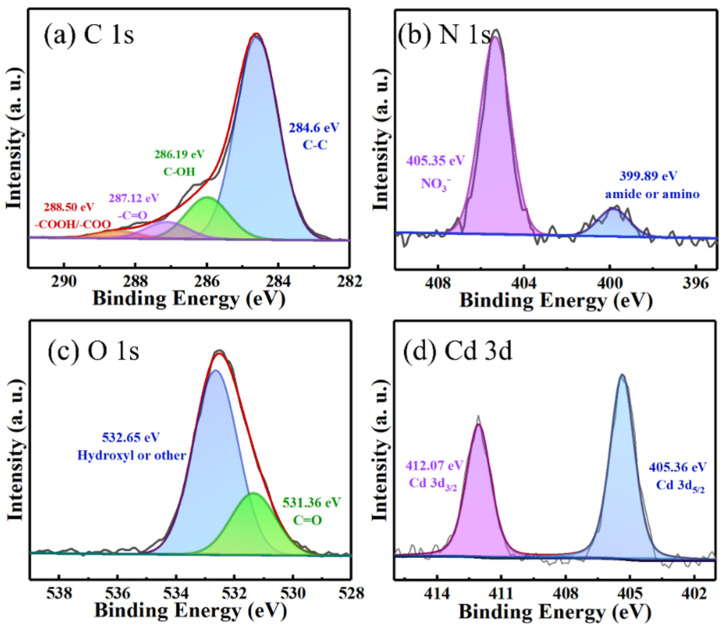
XPS spectra of (**a**) C 1s, (**b**) N 1s, (**c**) O 1s, and (**d**) Cd 3d for the LBC-P-1.3-200-2-Cd after adsorption of Cd(II). Batch adsorption experimental conditions: pH = 6.0, C_0_ = 100 mg∙L^−1^, m = 1.0 g∙L^−1^, v = 50 mL, T = 25 ± 1 °C, and t = 100 min.

To seek insight of the adsorption mechanism(s), XPS measurement was performed on the LBC-P-1.3-200-2 sample after the Cd(II) adsorption. Figure 9 showed the C 1s, O 1s, and N 1s core-level spectra of the Cd(II)-adsorbed LBC-P-1.3-200-2 sample. In comparison with the freshly-prepared biochar sample (Figure 5b), the deconvoluted C 1s spectrum demonstrated that the area of C−O (286.19 eV) and −C=O (287.12 eV) decreased by circa 2.5% and 1.0%, respectively (Figure 9a). This result was probably caused by the interactions between Cd(II) and carboxyl and carbonyl groups. The curve-fitted N 1s spectrum displayed the amide/amino (399.9 eV) and NO^3−^ (405.35 eV) bonds (Figure 9b). Compared with the state before adsorption (Figure 5c), the peak area of amide or amino groups decreased by circa 86.3%, which was due to their participating during the adsorption process. Moreover, the decreased intensity of O 1s XPS spectrum for the C−O was also observed after Cd(II) adsorption (Figure 9c). The area of peak corresponding to C=O decreased by 14.5%. It was found that the carboxylate groups (COO^−^) on the LBC-P-1.3-200-2 could easily adsorb Cd(II) through electrostatic interaction and ion exchange [56]. The curve-fitted Cd 3d core-level spectrum displayed the Cd(II) complexes at the peaks of 405.36 and 412.07 eV (Figure 9d), indicating that the surface groups were bound to Cd(II) successfully. According to the XPS results, the part of possible reaction equations were proposed as

R−COO−CH_3_ + Cd(II)−X → R−COO−Cd(II) −X + CH_3_^+^ (2)

R−COOH + OH^−^+ Cd(II)−X ↔ R−COO−Cd(II) −X +H_2_O (3)

R−O^−^ + Cd(II)−X ↔ R−O−Cd(II)−X (4)

R−NH_2_ + Cd(II)−X → R−NH_2_−Cd(II)−X (5)

where R represented other components of the biochar, Cd(II)−X represented Cd(II) species.

**Figure 10:**
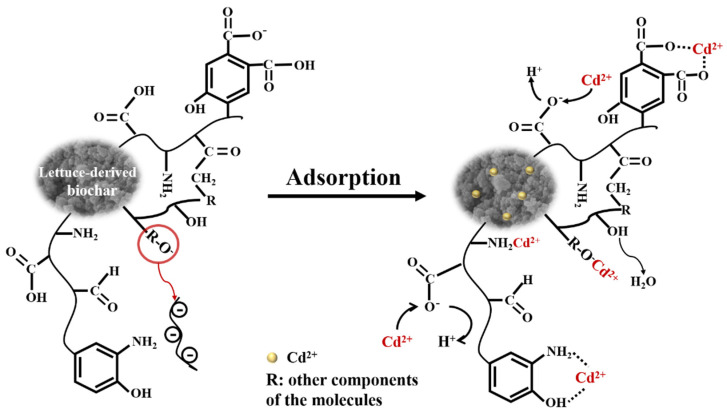
The postulated adsorption mechanisms of Cd(II) ions on the LBC-P-1.3-200-2 biochar sample.

To combine the above XPS characterization, zeta potentials, and adsorption performance results, the posted adsorption mechanisms of Cd(II) on the LBC-P-1.3-200-2 sample was proposed as shown in Figure 10. The interactions between the LBC-P-1.3-200-2 sample and Cd(II) were involved in the following: (i) the ions exchange took place between the protons in the adsorbing groups and Cd(II); (ii) the electrostatic attraction between COO^−^ and Cd(II) species; and (iii) the N atoms in amide or amino could donate electrons to form covalently bond or coordination complexes with Cd(II).

## 3. Materials and Methods

### 3.1. Materials

All chemical reagents, including phosphoric acid (H_3_PO_4_, 85%), cadmium nitrate tetrahydrate (Cd(NO_3_)_2_·4H_2_O), lead nitrate (Pb(NO_3_)_2_), zinc nitrate hexahydrate (Zn(NO_3_)_2·_6H_2_O), copper nitrate trihydrate (Cu(NO_3_)_2_·3H_2_O), nitric acid (HNO_3_), and sodium hydroxide (NaOH) were purchased from Kelong Chemical Reagent Co. (Chengdu, China), and were used as received unless specifically stated. The raw lettuce wastes were collected from Wenxing Town vegetable market near Jiangan Campus of Sichuan University (Chengdu, China). The deionized water (18.25 MΩ·cm) was used in the whole experimental process, which was purified from an ZYCGF−I−40L ultrapure reverse osmosis system (Sichuan Zhuoyue Water Treatment Equipment Co., Chengdu, China).

### 3.2. Hydrothermal Synthesis of Biochar without Activator Addition

The biochar samples were prepared by hydrothermal synthesis method with lettuce vegetation wastes as raw materials. Typically, the deionized water was used to wash impurities of lettuce waste and dried at 105 °C for 24 h, crushed and stored in a sealed container. Then, 2.5 g of dried lettuce waste was mixed with 50 mL of deionized water solution for 2 h. The mixed solution was transferred to a 100 mL PPL-lined autoclave (Enotai Instruments and Equipment Co., Beijing, China) to react at 230 °C for 2 h, and then cooled down to room temperature. The resultant biochar was removed out and filtered with qualitative filter paper. The solids retained by the filter paper were soaked in 1 mol·L^−1^ NaOH solution for 2 h to remove the impurity and tar. Then using a large amount of deionized water to wash the biochar until the pH was neutral. The resultant sample was dried at 60 °C for 12 h in an oven, then grinded in an agate mortar and the as-fabricated adsorbent was labeled as LBC-230-2 (L = lettuce waste, BC = biochar, hydrothermal temperature = 230 °C and time = 2 h).

### 3.3. Hydrothermal Synthesis of Biochar with H_3_PO_4_ Activating Agent

The single-step preparation process of the H_3_PO_4_-activated biochar was schematically illustrated in Scheme 1. Briefly, an aliquot 2.5 g of dried lettuce waste raw material was dissolved in a 50 mL H_3_PO_4_ solution with a certain concentration (Table S2) and continuously stirred for 2 h at room temperature (25 °C). The mixture was poured into the autoclave for hydrothermal treatment at different hydrothermal reaction times (1–6 h) (Table S1) and hydrothermal temperatures (170–260 °C) (Table S3), after which the subsequent steps were exactly the same as those used for the preparation of LBC-230-2. Adsorption performance of Cd(II) adsorption testing was designed to determine optimal reaction conditions of the activator concentration, hydrothermal reaction time, and temperature. The biochar produced by the addition of phosphoric acid was named as LBC-P. The biochar prepared under different hydrothermal conditions was marked as X-Y-Z, where X refers to the concentration of the activating agent; Y and Z refer to hydrothermal reaction temperature and reaction time, respectively. For example, LBC-P-1.3-200-2 represented the lettuce waste-based biochar prepared by adding 50 mL phosphoric acid solution of 1.3 mol·L^−1^ at hydrothermal reaction temperature of 200 °C and hydrothermal reaction time of 2 h.

### 3.4. Characterization

The FT-IR spectra of the as-fabricated biochar were collected on a Spectrum Two L1600300 spectrometer (PerkinElmer Inc., Waltham, MA, USA). The Raman spectra of the biochar were recorded on a DXR Raman Microscope Laser Raman spectrometer (Thermo Fisher Scientific Inc., Waltham, MA, USA). The XRD patterns of all samples were performed on a MiniFlex600 diffractometer (Rigaku Corporation, Tokyo, Japan) in a range of 10° to 80°. The surface chemical compositions of the as-fabricated biochar were determined by an Escalab 250Xi XPS (Thermo Fisher Scientific Inc., Waltham, MA, USA) characterization. The STA-449F3 thermal balance analyzer (NETZSCH Inc., Selb, Bavaria, Germany) was used to obtain the TG curves of the as-fabricated biochar. The surface morphology of biochars were recorded by SEM images on a REGULUS 8230 SEM (Hitachi Co., Tokyo, Japan) and TEM images on a FEI Tecnai G2 F20-TWIN TEM (FEI Co., Hillsboro, OR, USA). The micromeritics TRISTAR II3020 surface area analyzer (Micromeritics Instrument, Norcross, GA, USA) was used to determine the BET surface area of biochars. Zeta potentials of different pH solutions were recorded on a Zetasizer Nano ZS90 zeta potential analyzer (Malvern Panalytical, Malvern, UK). The total amount of heavy metal ions was determined by an ICP-OES (ICAP7400, BRE0002948, Thermo-Fisher Scientific Inc., Waltham, MA, USA).

### 3.5. Batch Adsorption Experiments

To study the adsorption behaviors of Cd(II) on the as-fabricated biochar, the batch adsorption experiments were carried out under different adsorption conditions. Detailed procedures (S1.2–S1.4) of the different adsorption conditions, such as effects of solution pH, initial concentration of Cd(II) ions, adsorbent dosage and existed heavy metal ions. During the batch adsorption experiment, a predetermined amount of biochar and 50 mL of solution with different Cd(II) initial concentrations were introduced in a 100 mL double-jacketed beaker, which then was placed on the adsorption apparatus and stirred magnetically for 100 min to reach adsorption equilibrium. The corresponding amount of Cd(NO_3_)_2_∙4H_2_O was dissolved in deionized water to prepare Cd(II) solution with an initial concentration of 40–1100 mg∙L^−1^. At a predetermined time interval, samples (each = 3 mL) were taken from the beaker and passed through a 0.22 μm membrane, and the concentrations of Cd(II) ions in the filtrate were measured by an ICP-OES. As shown in S1.5, adsorption experiments of the LBC-P-1.3-200-2 biochar towards Pb(II), Zn(II), Cu(II), and Cd(II) ions were also conducted to determine the effect of coexisted heavy metal ions. The amounts of heavy metal ions adsorbed on the biochar at adsorption equilibrium (*q_e_*) and the removal efficiency (*η*) can be calculated by the equations

(6) $$q_{e}=\frac{\left(c_{0}-c_{e}\right)V}{m}$$

(7) $$\eta =\frac{\left(c_{0}-c_{e}\right)\times 100\%}{c_{0}}$$

where *C_0_* (mg∙L^−1^) and *C_e_* (mg∙L^−1^) are the initial and equilibrium concentrations of heavy metals in the solution, *v* (L) is the volume of solution, and *m* (g) is the biochar dosage. Detailed experimental sections of adsorption kinetics, isotherms, and thermodynamics were described in S1.6–S1.8, respectively.

## 4. Conclusions

In summary, single-step hydrothermal synthesis of biochar from H_3_PO_4_-activation of lettuce waste was successfully accomplished in order to avoid traditional high-temperature pyrolysis. The optimal reaction conditions of hydrothermally synthesized biochar were systematically explored to adjust the physicochemical properties and microstructure of biochar for high adsorption performance. The as-fabricated biochar delivered an excellent adsorption performance of 195.8 mg∙g^−1^ with Cd(II) from aqueous solution with a rapid adsorption rate, the adsorption kinetics demonstrated to be consistent with the PSO model, and the adsorption equilibrium could be attained within 30 min. The good fitting of adsorption isotherms by Sips model revealed the adsorption of Cd(II) on the heterogeneous surfaces of the as-fabricated biochar. The thermodynamic results revealed an spontaneous and exothermic adsorption process of Cd(II) on the biochar samples. The postulated adsorption mechanisms were corresponding to ion exchange, electrostatic attraction, and surface complexation. This work offers a novel strategy for producing cheap and promising carbon-based adsorbents for absorbing heavy metals in aquatic environments and may also provide a new avenue for sustainable management of waste biomass in the future.

## Figures and Tables

**Table 1:** Kinetic parameters for adsorption of Cd(II) onto LBC-P-1.3-200-2 at an initial Cd(II) concentration of 100 mg∙L^−1^.

**Samples**	**Models**		**Model Parameters**			
			***k_f_* (min^−1^)**	***k_s_* (g∙mg^−1^·min^−1^)**	***q_max_* (mg∙g^−1^)**	***R* ^2^**
LBC-P-1.3-200-2	PFO		0.331	−	87.40	0.988
	PSO		−	0.008	91.75	0.999
**Samples**	**Models**		**Model Parameters**			
			***k_id_* (mg·g^−1^·min^−0.5^)**	***C_id_* (mg∙g^−1^)**	***k_f_* (min^−1^)**	***R* ^2^**
LBC-P-1.3-200-2		1	31.76	2.808	−	0.955
	Weber–Morris	2	4.443	66.49	−	0.910
		3	0.406	85.95	−	0.905
	Boyd		−	−	0.071	0.950

**Table 2:** Isotherm parameters for the adsorption of Cd(II) onto LBC-P-1.3-200-2.

Isotherms	Parameters	Temperature (K)			
		288.15	298.15	308.15	318.15
Langmuir	*q_m_* (mg∙g^−1^)	171.5	173.2	142.6	115.4
	*K_L_* (L∙mg^−1^)	0.092	0.062	0.076	2.290
	*R* ^2^	0.612	0.560	0.398	0.615
Freundlich	*K_F_* (mg^1−1/n^∙L^1/n^∙g^−1^)	60.08	65.19	58.84	55.46
	1/n	0.170	0.153	0.139	0.133
	*R* ^2^	0.976	0.945	0.929	0.920
Temkin	*K_T_* (L∙mg^−1^)	77.07	633.1	452.1	246.5
	*b_t_*	150.9	193.7	232.8	247.3
	*R* ^2^	0.911	0.847	0.914	0.878
Sips	*q_m_*	195.9	195.8	159.1	144.3
	*K_s_*	0.199	0.028	0.038	0.094
	*n_S_*	0.082	0.008	0.009	0.023
	*R* ^2^	0.989	0.987	0.971	0.965

**Table 3:** Comparison of the maximum adsorption capacities of heavy metals onto various adsorbents.

Adsorbents		Heavy Metals	Reaction Temperature (°C)	*q_m_* (mg∙g^−1^)	Ref.
Raw Materials	Activating Agents				
Lettuce waste	−	Cd(II)	25	20.5	This work
Lettuce waste	H_3_PO_4_	Cd(II)	25	198.5	This work
Bagasse pitch	H_2_SO_4_	Cd(II)	25	38.3	[44]
Chicken feather	H_3_PO_4_	Cd(II)	25	7.8	[45]
*Pennisetum* sp. straw	KMnO_4_, Fe(NO_3_)_3_	Cd(II)	25	95.2	[46]
Microfibrillated cellulose	Succinic anhydride	Cd(II)	25	236.1	[47]
Phragmites australis	Urea phosphate	Cd(II)	30	40.7	[48]
Apricot stones	H_2_SO_4_	Cd(II)	25	33.6	[49]
Olive stone	ZnCl_2_	Cd(II)	30	1.7	[50]
Swine manure	MnO_2_	Cd(II)	25	45.8	[51]
Coffee grounds	-	Cd(II)	20	15.7	[52]

**Table 4:** Thermodynamic parameters for the adsorption of Cd(II) onto LBC-P-1.3-200-2.

C_0_ (mg∙L^−1^)	ΔH° (kJ∙mol^−1^)	ΔS° (J∙mol^−1^∙K^−1^)	ΔG° (kJ∙mol^−1^)			
			288.15 K	298.15 K	308.15 K	318.15 K
100	−18.88	−46.97	−5.35	−4.88	−4.41	−3.94
200	−14.93	−39.44	−3.57	−3.17	−2.78	−2.38

## Data Availability

Data are contained within the article.

## Supplementary Materials

The following supporting information can be downloaded online. Determination of optimal preparation conditions of different types of biochar (S1.1), effect of environmental factors on adsorption performance. (S1.2–S1.4), effect of existed heavy metal ions (S1.5), adsorption kinetics, isotherms and thermodynamics (S1.6–S1.8) [54]; FTIR, XRD, Raman, and TG curves of the H_3_PO_4_-activated biochar at different reaction time and activator concentration (Figures S1 and S2), the N_2_ adsorption–desorption isotherms of LBC-230-2 obtained without H_3_PO_4_ activation during hydrothermal synthesis (Figure S3), adsorption capacity for different heavy metal ions onto LBC-P-1.3-200-2 (Figure S4a), the pseudo-first-order (PFO) model fitted adsorption kinetics for different heavy metal ions on LBC-P-1.3-200-2 (Figure S4b), the Langmuir and Temkin isotherm models-fitted curves of Cd(II) adsorbed on the LBC-P-1.3-200-2 at different adsorption temperatures (Figure S4c,d). Different hydrothermal synthesis conditions for H_3_PO_4_-activated biochar (Tables S1–S3), BET characterization results of biochar samples (Table S4), the element content distribution of LBC-P-1.3-200-2 in XPS spectra (Table S5), fitted kinetic parameters for different heavy metal ions on the LBC-P-1.3-200-2 (Table S6).

## Sample Availability

The samples are available from the authors.
