# Supplementary material for: Single-Step Hydrothermal Synthesis of Biochar from H3PO4-Activated Lettuce Waste for Efficient Adsorption of Cd(II) in Aqueous Solution
Source: Molecules. 2022 Jan 2;27(1):269. doi: 10.3390/molecules27010269 (PMC8746578; doi:10.3390/molecules27010269)
Supplement: Supplementary file 1 [file molecules-27-00269-s001.zip › molecules-1469455-supplementary.pdf]

Supporting Information

Single-step Hydrothermal Synthesis of Biochar from H_3PO_4-Activated Lettuce Waste for Efficient Adsorption of Cd(II) in Aqueous Solution

Quyun Chen [a], Tian Cheng Zhang,[b] Like Ouyang,[a] Shaojun Yuan [a,*]

[a] Low-carbon Technology & Chemical Reaction Engineering Lab, College of Chemical Engineering, Sichuan University, Chengdu 610065, P. R. China.
[b] Civil & Environmental Engineering Department, University of Nebraska-Lincoln, Omaha, NE 68182-0178, USA
*Corresponding author: E-mail: ysj@scu.edu.cn; Tel/Fax: +86-28-85405201

S1 Supplementary Experimental

S1.1 Determination of optimal preparation conditions of different types of biochars. In this study, different types of the biochars were prepared under different preparation conditions, as shown in detail in Tables S1 to S3. Batch adsorption experiments were conducted to evaluate the effect of activator concentration, hydrothermal reaction time and hydrothermal reaction temperature on the adsorption capacities of as-prepared biochar toward Cd(II) ions. The batch experimental conditions were controlled as follows: pH = 6 ± 0.05, T = 25 °C, C_0 = 100 mg·L^{-1}, m = 0.05 g, V = 0.05 L, t = 100 min.

The determination of optimal preparation conditions was performed as shown in Tables S1-S3. To use H_3PO_4 solution as the activation agent, the optimal hydrothermal time at 2 h was determined as shown in Fig. 1a (see Table S1), the optimal concentration of H_3PO_4 was determined at 1.3 mol·L^{-1} as shown in Fig. 1b (see Table 2), and the optimal hydrothermal temperature was determined at 200 °C as shown in Fig. 1c (see Table 3) for the biochar.

S1.2 Effect of solution pH. In the experiment, the pH value of the solution was adjusted with 0.1 M HNO_3 and 0.1 M NaOH, respectively. Experimental conditions: $C_0 = 100$ mg·L^{-1}, m = 0.05 g, V = 0.05 L, T = 25 ± 1 °C, and t = 100 min.

S1.3 Effect of Cd(II) initial concentration. Changed the Cd(II) initial concentrations as 40−1100 mg·L^{-1}. Experimental conditions: pH = 6.0 ± 0.05, m = 0.05 g, V = 0.05 L, t = 100 min and T = 25 ± 1 °C.

S1.4 Effect of LBC-P-1.3-200-2 dosage. To test of the effect of the LBC-P-1.3-200-2 dosage, each of the different adsorbent dosages was added to 50 mL of the Cd(II) solution. Experimental conditions: $C_0 = 100$ mg·L^{-1}, pH = 6.0 ± 0.05, t = 100 min and T = 25 ± 1 °C.

S1.5 Effect of existed heavy metal ions. Applicability of LBC-P-1.3-200-2 for Cd(II), Cu(II), Zn(II) and Pb(II) removal were studied using batch experiments in a reaction mixture of 0.05 g of adsorbent and 0.05 L of metal solution containing each of the heavy metals at 5 mmol·L^{-1}. The result was shown in Fig. S3a. Experimental conditions: $C_0 = 5$ mmol·L^{-1}, m = 0.05 g, V = 0.05 L, pH = 6.0 ± 0.05, t = 100 min and T = 25 ± 1 °C.

S1.6 Adsorption kinetics. The time that reach adsorption equilibrium for the adsorption experiment was determined by kinetic tests with Cd(II) at t = 0−100 min using different types of biochar at the test conditions of: $C_0 = 100$ mg·L^{-1}, pH = 6.0 ± 0.05, m = 0.05 g, V = 0.05 L, and T = 25 ± 1 °C. In order to study the effect to remove other heavy metal ions, adsorption kinetic studies were carried out under optimal conditions for Cd(II), Cu(II), Zn(II) and Pb(II) with LBC-P-1.3-200-2 at conditions of: pH = 6 ± 0.05, T = 25 ± 1 °C, $C_0 = 5$ mmol·L^{-1}, m = 0.05 g, V = 0.05 L, t = 0−100 min.

The pseudo-first-order (PFO) and pseudo-second-order (PSO) kinetic models are utilized to seek the underlying mechanism of the adsorption process. Lagergren[1] proposed that the

pseudo-first order model can be used to describe the liquid-solid phase adsorption system, while Ho and McKay[2] proposed that the pseudo-second-order model describes a kinetic process of the adsorption of divalent metal ions on solid adsorbents. The data of kinetic studies were fitted with the PFO model[1] (Eq. 1) and the PSO model[2] (Eq. 2):

$$c = c_0 \cdot e^{(-k_f t)} \tag{1}$$

$$q_t = \frac{k_s \cdot q_e^2 \cdot t}{1 + k_s \cdot q_e \cdot t} \tag{2}$$

where q_e and q_t (mg·g^{-1}) are the amounts of heavy metals adsorbed at equilibrium and at time t (min); k_f (min^{-1}) is the rate constant of the pseudo-first-order model; k_s (g· mg^{-1}·min^{-1}) is the rate constant of pseudo-second order model.

The Weber-Morris model was commonly used to confirm whether the intraparticle diffusion was the rate-controlling step, while the Boyd model was applicable to determine the contribution of the boundary layer or film diffusion. The data of kinetic studies were fitted with the Weber-Morris intra-particle diffusion model[3] (Eq. 3), and the Boyd model[4] (Eq. 4):

$$q_t = k_{pi} \cdot t^{\frac{1}{2}} + C \tag{3}$$

$$-ln(1\text{-}F) = k_f \cdot t \tag{4}$$

where k_{pi} (mg·g^{-1}·min$^{-0.5}$) is the diffusion rate constant of intra-particle diffusion kinetic model; F is the fractional attainment of equilibrium calculated from the ratio of q_t/q_e; and k_f is the film diffusion rate constant (min^{-1}).

S1.7 Adsorption isotherms. Adsorption isotherms studies were carried out under optimal conditions for Cd(II) with LBC-P-1.3-200-2 at conditions of: pH = 6 ± 0.05, m = 0.05 g, V = 0.05 L, t = 100 min, T = 15−45 °C and C_0 = 40−1100 mg·L^{-1}. The adsorption isotherms are frequently fitted by different isotherm models, such as Langmuir, Freundlich, Temkin and Sips isotherm models:

$$q_e = \frac{K_L \cdot q_m \cdot C_e}{1 + K_L \cdot C_e} \tag{5}$$

$$q_e = K_F C_e^{1/n} \tag{6}$$

$$q_e = A \cdot lnK_T + A \cdot lnC_e \tag{7}$$

$$q_e = \frac{q_m \cdot K_S \cdot C_e^{1/n_s}}{1 + K_S \cdot C_e^{1/n_s}} \tag{8}$$

where C_e ($mg \cdot L^{-1}$) is the equilibrium concentration after adsorption; q_e ($mg \cdot g^{-1}$) is the adsorption capacity for Cd(II) adsorbed at equilibrium; K_L ($L \cdot mg^{-1}$) is the Langmuir adsorption constant; q_m ($mg \cdot g^{-1}$) is the maximum adsorption capacity calculated by the Langmuir model; K_F and n are Freundlich constants; n represents the degree of favorable adsorption process; K_F ($mg^{1-1/n} \cdot L^{1/n} \cdot g^{-1}$) is the adsorption capacity of the adsorbent; $A = RT / b_t$, where R (8.314 $J \cdot mol^{-1} \cdot K^{-1}$) is the gas constant; T (K) is the absolute temperature; b_t is the Temkin constant related to the heat of adsorption ($kJ \cdot mol^{-1}$); K_S ($L \cdot g^{-1}$) is the Sips model constant; n_s is the Sips model index.

S1.8 Adsorption thermodynamics. Adsorption thermodynamics studies were carried out under optimal conditions for Cd(II) with LBC-P-1.3-200-2 at conditions of: pH = 6 ± 0.05, m = 0.05 g, V = 0.05 L, t = 100 min, C_0 = 100 $mg \cdot L^{-1}$ and T = 15−45 °C. The changes in enthalpy ($\Delta H°$), entropy ($\Delta S°$) and Gibbs free energy ($\Delta G°$) were calculated with Eqs. (9−11)[5] to determine the thermodynamic properties:

$$K = \frac{q_e}{C_e} \tag{9}$$

$$\Delta G° = -RT\ln K \tag{10}$$

$$lnK = \frac{\Delta S°}{R} - \frac{\Delta H°}{RT} \tag{11}$$

where K ($L \cdot g^{-1}$) is the equilibrium constant; q_e ($mg \cdot g^{-1}$) is the adsorption capacity; C_e ($mg \cdot L^{-1}$) is the equilibrium Cd(II) concentration; R (8.314 $J \cdot mol^{-1} \cdot K^{-1}$) is the general gas constant; and T (K) is the temperature.

S2 Supplementary Results

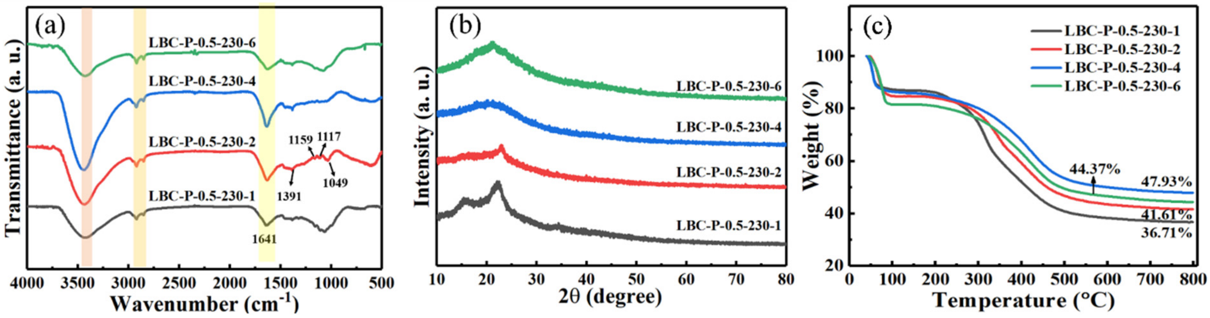

Figure S1. (**a**) FT-IR spectra, (**b**) XRD patterns, and (**c**) TG curves of LBC-P-0.5-230-*X* (*X* = 1, 2, 4, 6 h) samples at different reaction time.

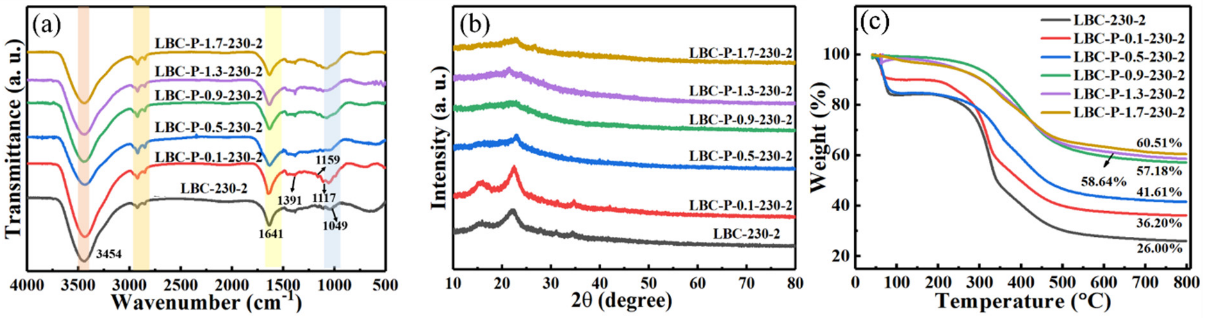

Figure S2. (**a**) FT-IR spectra, (**b**) XRD patterns, and (**c**) TG curves of LBC-P-*X*-230-2 (*X* = 0, 0.1, 0.5, 0.9, 1.3, 1.7 mol·L^{-1}) samples with different activator concentration.

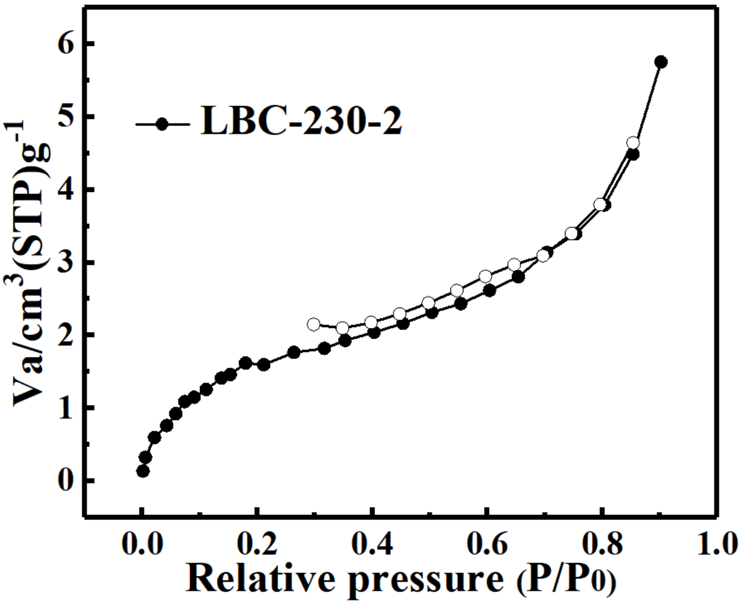

Figure S3. The N_2 adsorption-desorption isotherms of LBC-230-2 obtained without H_3PO_4 activation during hydrothermal synthesis.

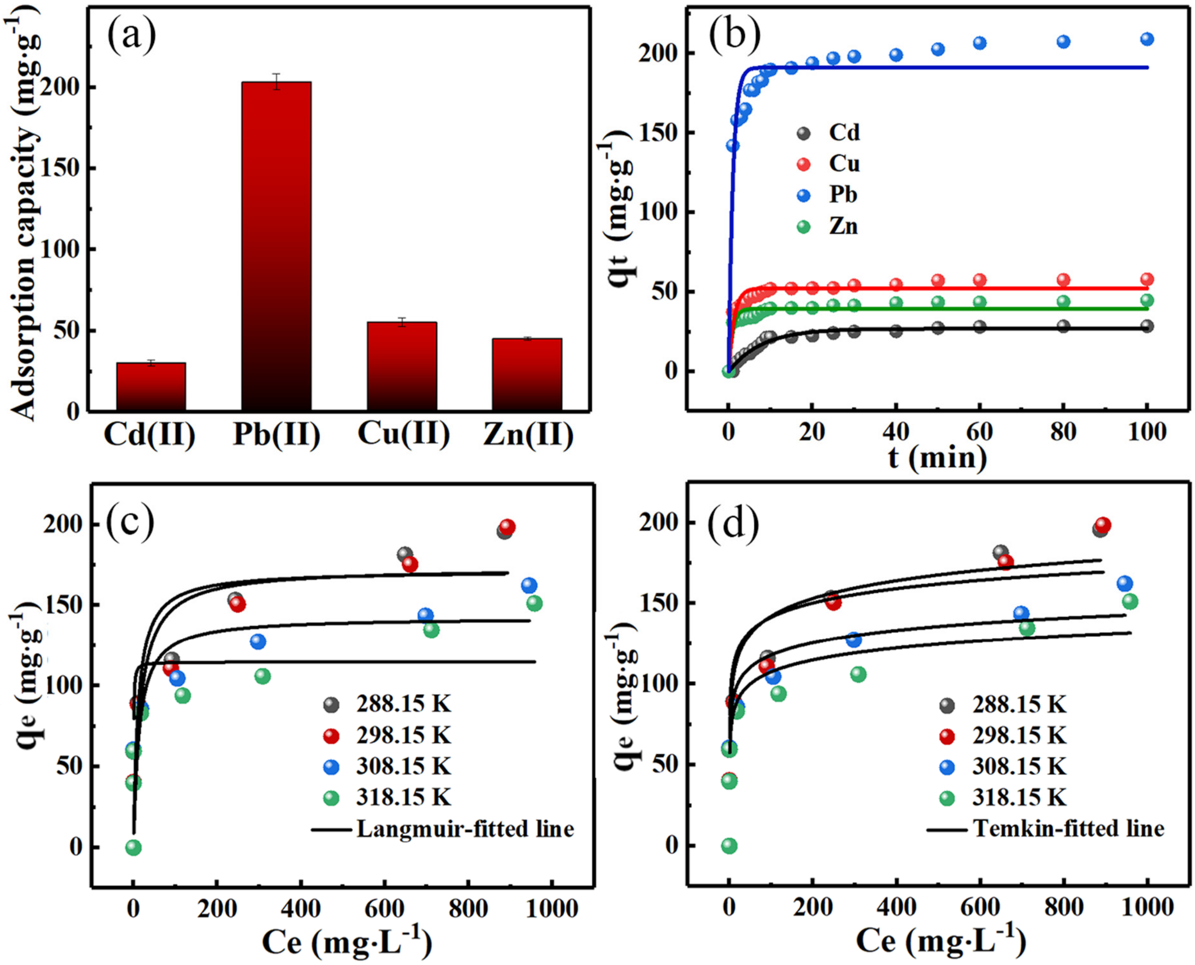

Figure S4. (**a**) Adsorption capacity of LBC-P-1.3-200-2 for Cd(II), Cu(II), Zn(II), and Pb(II). (**b**) Adsorption kinetics of LBC-P-1.3-200-2 for Cd(II), Cu(II), Zn(II), and Pb(II) and fitting curves of the pseudo-first order model. (**c**) Langmuir- and (**d**)Temkin-model fitted adsorption isotherms of Cd(II) ions on the LBC-P-1.3-200-2 at different adsorption temperature.
s of 288.15, 298.15, 308.15 and 318.15K. Experimental conditions: pH = 6.0, C_0 = 100 mg·L^{-1}, m = 0.05 g, V = 0.05 L, t = 100 min.

S3 Supplementary Tables

Table S1. Different reaction time for hydrothermal synthesis of H_3PO_4-activated biochars

Samples	Reaction time	Activator types	Activator concentration	Reaction temperature
LBC-P-0.5-230-1	1 h	H_3PO_4	0.5 mol·L^{-1}	230 °C
LBC-P-0.5-230-2	2 h	H_3PO_4	0.5 mol·L^{-1}	230 °C
LBC-P-0.5-230-4	4 h	H_3PO_4	0.5 mol·L^{-1}	230 °C
LBC-P-0.5-230-6	6 h	H_3PO_4	0.5 mol·L^{-1}	230 °C

Table S2. Different activator concentrations for hydrothermal synthesis of H_3PO_4-activated biochars

Samples	Reaction time	Activator types	Activator concentration	Reaction temperature
LBC-P-0.1-230-2	2 h	H_3PO_4	0.1 mol·L^{-1}	230 °C
LBC-P-0.5-230-2	2 h	H_3PO_4	0.5 mol·L^{-1}	230 °C
LBC-P-0.9-230-2	2 h	H_3PO_4	0.9 mol·L^{-1}	230 °C
LBC-P-1.3-230-2	2 h	H_3PO_4	1.3 mol·L^{-1}	230 °C
LBC-P-1.7-230-2	2 h	H_3PO_4	1.7 mol·L^{-1}	230 °C

Table S3. Different reaction temperatures for hydrothermal synthesis of H_3PO_4-activated biochars

Samples	Reaction time	Activator types	Activator concentration	Reaction temperature
LBC-P-1.3-170-2	2 h	H_3PO_4	1.3 mol·L^{-1}	170 °C
LBC-P-1.3-200-2	2 h	H_3PO_4	1.3 mol·L^{-1}	200 °C
LBC-P-1.3-230-2	2 h	H_3PO_4	1.3 mol·L^{-1}	230 °C
LBC-P-1.3-260-2	2 h	H_3PO_4	1.3 mol·L^{-1}	260 °C

Table S4. BET characterization results of different biochars obtained without and with the addition of H_3PO_4 activator

Sample	S_{BET} [m^2·g^{-1}]	Vtotal [cm^3·g^{-1}]	D [nm]
LBC-230-2	6.96	0.009	5.12
LBC-P-1.3-200-2	5.20	0.018	6.20

Table S5. The elemental content distribution of LBC-P-1.3-200-2 in XPS spectra

Name	C 1s	O 1s	N 1s	P 2p
LBC-P-1.3-200-2	79.19%	17.38%	2.38%	0.62%

Table S6. Fitted kinetic parameters of different heavy metal ions adsorbed on the LBC-P-1.3-200-2 sample.

Kinetic Modles	Parameters	Cd(II)	Cu(II)	Pb(II)	Zn(II)
Pseudo-first-order parameters	$q_{e,exp}$ (mg·g^{-1})	28.50	57.98	209.0	44.71
	$q_{e,cal}$ (mg·g^{-1})	26.94	52.34	191.2	39.51
	k_f(min^{-1})	0.128	0.774	1.007	1.035
	R^2	0.971	0.882	0.916	0.843
Pseudo-second-order parameters	$q_{e,exp}$ (mg·g^{-1})	28.50	57.98	209.0	44.71
	$q_{e,cal}$ (mg·g^{-1})	31.00	55.58	201.3	42.14
	k_s (g·mg^{-1}·min^{-1})	0.540	1.477	1.771	1.933
	R^2	0.969	0.967	0.981	0.942

References

1. Lagergren, S. K., About the theory of so-called adsorption of soluble substances. *Sven. Vetenskapsakad. Handingarl* **1898,** *24*, 1-39.

2. Ho, Y. S.; McKay, G., Pseudo-second order model for sorption processes. *Process biochemistry (1991)* **1999,** *34* (5), 451-465.

3. Weber Morris, W., Kinetics of adsorption on carbon from solution. *Proc. Amer. Soc. civ. Engrs* **1963,** *89*, 31-59,3483.

4. Yuan, S.; Zhang, P.; Yang, Z.; Lv, L.; Tang, S.; Liang, B., Successive grafting of poly(hydroxyethyl methacrylate) brushes and melamine onto chitosan microspheres for effective Cu(II) uptake. *Int J Biol Macromol* **2018,** *109*, 287-302.

5. Ghorbani, F.; Kamari, S.; Zamani, S.; Akbari, S.; Salehi, M., Optimization and modeling of aqueous Cr(VI) adsorption onto activated carbon prepared from sugar beet bagasse agricultural waste by application of response surface methodology. *Surfaces and Interfaces* **2020,** *18*.
